# Lung transplant list withdrawal in a liver transplant patient thanks to elexacaftor-tezacaftor-ivacaftor: a case report

**DOI:** 10.1186/s13052-024-01713-x

**Published:** 2024-07-30

**Authors:** Arianna Traunero, Anna Galletti, Sergio Ghirardo, Egidio Barbi, Massimo Maschio

**Affiliations:** 1 Department of Surgical, Medical and Health Sciences, University of Trieste, Via dell’Istria 65/1, Trieste, 34127 Italy; 2 Institute for Maternal and Child IRCCS Burlo Garofolo, Trieste, Italy

**Keywords:** Cystic fibrosis, Elexacaftor-tezacaftor-ivacaftor, Liver transplant, Advanced lung disease

## Abstract

**Background:**

Elexacaftor-tezacaftor-ivacaftor (ETI) is a transmembrane conductance regulator modulator that significantly improves lung function in patients affected by cystic fibrosis (CF). This triple drug is currently not indicated in liver transplant patients, as clinical trials including subjects with previous solid organ transplantation are lacking.

**Case presentation:**

We report on a liver transplant girl with CF-related advanced pulmonary disease meeting clinical criteria for lung transplant, who started the triple modulator because she could not get on the lung transplant waiting list due to psycho-social motivations. Since initiation of ETI therapy, she has experienced a significant improvement in respiratory function and quality of life, without adverse effects.

**Conclusions:**

This case shows that ETI therapy can represent a lifesaving drug for individuals without alternatives, even in liver transplant patients. The clinical benefits of the modulator overcome risks, which may be limited with a close drug monitoring of immunosuppressants serum levels and functional liver tests.

## Background

Elexacaftor-tezacaftor-ivacaftor (ETI) is a transmembrane conductance regulator modulator that has superior efficacy in patients affected by cystic fibrosis (CF) compared to previously approved modulators (lumacaftor-ivacaftor or tezacaftor-ivacaftor or ivacaftor alone). This triple drug combination causes considerable increases in CFTR-channel function, and it is currently recommended for patients $\:\ge\:$ 2 years with at least one copy of F508del mutation or with other rarer mutations ETI-responsive based on in vitro data [1].

ETI is associated with improved lung function, with a significant increase of the forced expiratory volume in the first second (FEV1) and reduced respiratory symptoms and lung exacerbations. Furthermore, this therapy provides several extrapulmonary effects, such as an improvement of body weight and body mass index (BMI), and some evidence suggests that it is also effective at delaying/reversing pancreatic insufficiency, reducing gastrointestinal symptoms, and improving glycemic control [2].

ETI is generally well tolerated without substantial adverse effects. However, this triple drug is not indicated in liver transplant patients, as clinical trials including subjects with previous solid organ transplantation are lacking. This aspect represents a considerable limitation, as severe hepatic disease regards 4.5–10% of individuals affected by CF, and liver transplant represents a valid option in case of complications such as portal hypertension or cirrhosis [3]. Recent reports suggest that ETI is safe in liver transplant patients, who can benefit from this drug, avoiding the specific and progressive deterioration of respiratory function [4–6].

We describe the substantial change of life perspective in a liver transplant girl with advanced lung disease who greatly benefited from ETI therapy. Despite meeting the organ criteria, she started the triple modulator because she could not get on the pulmonary transplant waiting list.

### Case presentation

**Fig. 1:**
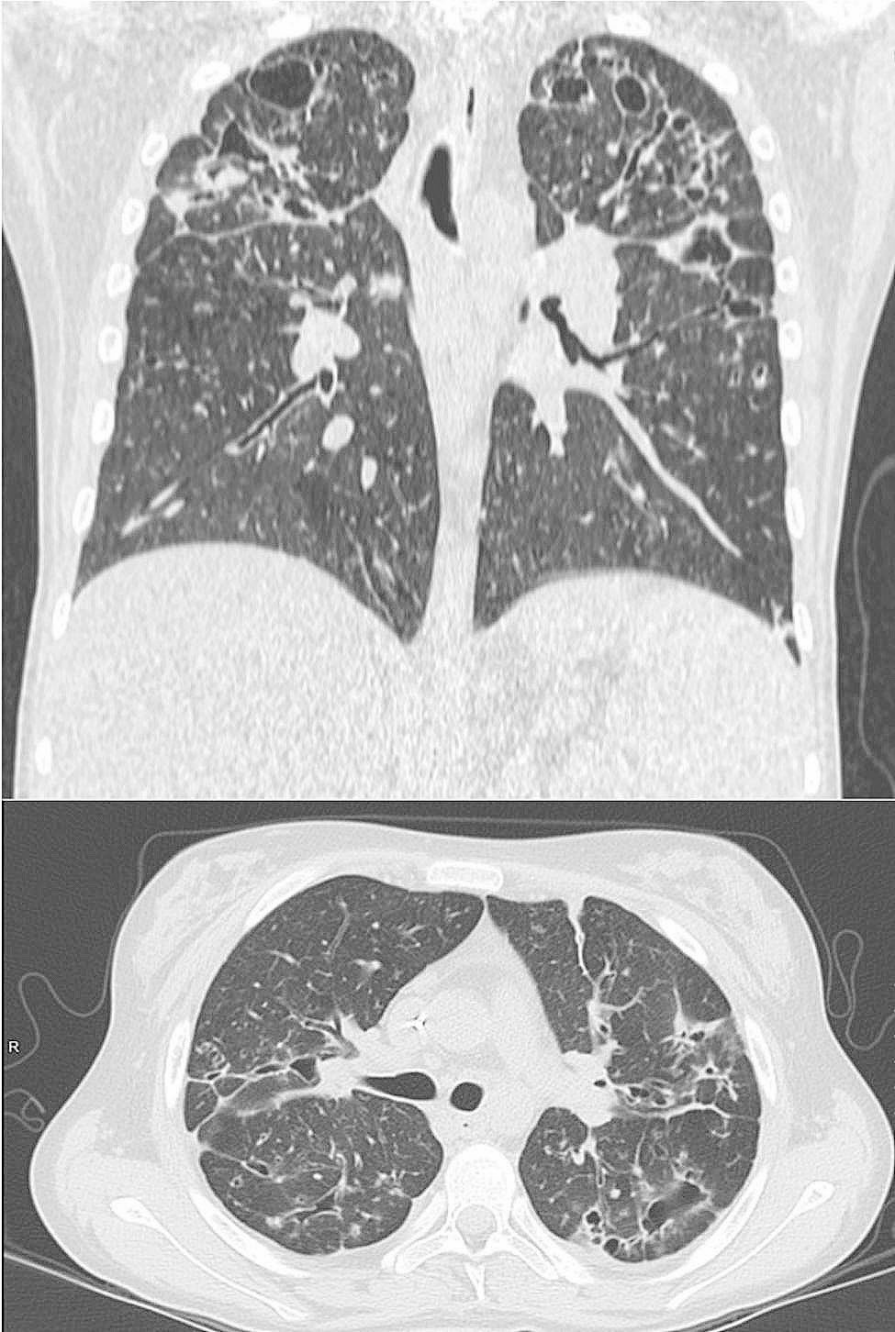
Chest CT-scans show multiple giant apical bronchiectasis and a middle lobe syndrome

We report on a 17-year-old girl affected by CF (homozygous for F508del mutation) with severe pulmonary and hepatic impairment. She presented chronic colonization by *methicillin-resistant Staphylococcus aureus* (MRSA) and *Pseudomonas aeruginosa*. She received a liver transplant in 2016, at the age of 11; however, her pulmonary function progressively deteriorated in the following 5 years. She underwent many hospitalizations for pulmonary exacerbations (10 episodes between 2020 and 2022), requiring intravenous multiple and prolonged antimicrobial therapy. In this period, her lung function showed fluctuating values of FEV1, between 40% and 50% of the predicted, with short-living recoveries after repeated intravenous antibiotic cycles. The combination of ivacaftor and tezacaftor was tested for 12 months with no significant clinical benefits. Due to the ongoing respiratory decline, characterized by deteriorating functional tests and progressive radiological images worsening, chest-CT scans showing multiple giant apical bronchiectasis and a middle lobe syndrome (Fig. 1), lung transplantation had been considered. However, the patient could not get on the pulmonary transplant waiting list because of severe social and phycological problems leading her to poor treatment adherence.

Regarding the hepatic function, the girl presented severe steatosis with normal levels of transaminases, compatible with a mild chronic liver rejection controlled by mycophenolate mofetil and tacrolimus.

In July 2022, she started ETI treatment at the recommended adult dose in an off-label regimen (specifically “class C non-negotiated” (Cnn) regimen, obtained with regional support). Since starting this therapy, she has never needed hospitalizations for lung exacerbations or pulmonary complications, and her FEV1 has increased by 12–15% within two months. This significant recovery has persisted over time, with a mean steady FEV1 of 62% after a year of treatment. She also significantly improved her nutritional status, achieving an ideal BMI (from 16 to 22 kg/m^2^). Remarkably, during the first year of ETI therapy, her liver function tests remained normal (reaching maximum bilirubin levels of 1.30 mg/dl, of which 0.37 mg/dl of direct bilirubin, and maximum gamma-glutamyl transferase levels of 81 U/l), as well as creatinine kinase values (mean of 30 U/l). Serum immunosuppressant concentrations remained stable without requiring dose adjustments.

## Discussion and conclusions

In CF-related advanced pulmonary disease, defined by an FEV1 lower than 40% of the predicted, ETI treatment, if indicated, is associated with rapid clinical improvement, often leading to suspension or withdrawal of the patient from the lung transplantation list. In an observational study, ETI administration for one to three months in 245 patients with advanced lung disease was associated with a mean increase in predicted FEV1 by 15.1%, which is consistent with our case, in addition to a substantial reduction in chronic oxygen therapy and non-invasive ventilation requirement [7]. Furthermore, this improvement is long-lasting, as demonstrated by another study, where among 65 patients eligible for a lung transplant, the improvement in FEV1 after ETI initiation remained stable after a mean follow-up of one year, allowing most individuals to be removed and remain off the transplant list [8].

Due to the possible risk of liver failure and, more frequently, an increase of transaminase and bilirubin, ETI treatment is currently not routinely indicated in individuals affected by CF with a previous liver transplantation. This typology of patients has not been included in clinical trials that evaluate safety and efficacy of ETI therapy.

A previous study reported that in a patient with cirrhosis and portal hypertension, ETI led to liver transplantation. Indeed, the drug’s pharmacokinetics implies several drug-to-drug interactions: the triple modulator is a substrate of CYP3A4, and ivacaftor also weakly inhibits P-glycoprotein, an enzyme that metabolizes several drugs, including immunosuppressants such as cyclosporine, everolimus, sirolimus and tacrolimus, thus elevating their serum concentrations. Therefore, ETI may cause further hepatotoxicity.

Our patient’s respiratory impairment was so severe that lung transplantation was considered the only reasonable option. However, she was not eligible for this treatment for psycho-social reasons due to poor compliance with treatments and therapies. On the other hand, she did not meet the criteria to start the triple modulator because she had previously received a hepatic transplant. We tried administering the triple modulator to improve her poor prognosis based on previous reports exploring the ETI regimen’s safety and tolerability in liver transplant patients with CF. Remarkably, several retrospective case series involving liver transplant individuals with an immunosuppressive regimen of drug therapy metabolized by P-glycoprotein showed that the ETI regimen is safe and leads to clinical benefits in lung function, quality of life and BMI. In these patients, a transient elevation in transaminase and bilirubin resolved in most cases after ETI dose reduction and elevations in serum concentration of tacrolimus were managed with dose adjustments of the immunosuppressive regimen [4–6].

During the one year of follow-up, our young patient maintained regular hepatic markers, with stable serum concentration of the immunosuppressants, and no dose modulation was required. Considering the clinical outcomes, she has experienced a significant improvement in respiratory function (with a stable increase of FEV1 of more than 12%), BMI and quality of life, with a remarkable change in her life perspective. These aspects have been also objectified by the relevant difference in scores obtained on the CFQR (Cystic Fibrosis Questionnaire-Revised), health-related quality of life questionnaire filled out by the patient before and after treatment (total average score of 60/100 and 78/100 points respectively).

Our case further indicates that ETI can be well tolerated in patients with previous liver transplantation. It represents a lifesaving drug for patients without alternatives, dramatically improving their quality of life. This report adds to the evidence suggesting that the clinical benefits of ETI in liver transplant patients overcome risks, which may be limited with a close drug monitoring of immunosuppressants serum levels and functional liver tests.

Further studies with a long-term follow-up are required to confirm the safety of this treatment in this specific group of patients.

## Data Availability

All data generated or analyzed during this study are included in this published article.

## Abbreviations

ETI: Elexacaftor-tezacaftor-ivacaftor
CF: Cystic fibrosis
FEV1: Forced expiratory volume in the first second
BMI: Body mass index
MRSA: Methicillin-resistant Staphylococcus aureus
CFQR: Cystic Fibrosis Questionnaire-Revised
